# Acute hemorrhagic leukoencephalitis following the first dose of BNT162b2 vaccine against SARS-CoV-2: A case report

**DOI:** 10.1016/j.heliyon.2024.e25545

**Published:** 2024-02-02

**Authors:** Konstantinos Kalafatakis, Anna Margoni, Maria-Eleni Liakou, Christos Stenos, Panagiotis Toulas, Penelope Korkolopoulou, Eleftheria Lakiotaki, Spiridon A. Lafazanos, Katerina Zekiou, Panagiota Kardara, Aspasia Terentiou, Georgios Nikolaou, Georgios Stouraitis

**Affiliations:** a Department of Neurology, 417 Army Share Fund Hospital, Athens, Greece; b Faculty of Medicine & Dentistry (Malta Campus), Queen Mary University of London, Victoria, Malta; c Research Unit of Radiology, Second Department of Radiology, Medical School, National and Kapodistrian University of Athens, Athens, Greece; d First Department of Pathology, LAIKON University Hospital, Medical School, National and Kapodistrian University of Athens, Athens, Greece; e Department of Neurosurgery, 417 Army Share Fund Hospital, Athens, Greece

**Keywords:** Acute hemorrhagic leukoencephalitis, BNT162b2 vaccine, Brain biopsy, Coma, Case report

## Abstract

Acute hemorrhagic leukoencephalitis (AHLE), is a rare inflammatory demyelinating disorder, variant of acute disseminated encephalomyelitis. The diagnosis of AHLE remains challenging due to the rarity of the disease and the lack of a reliable biomarker. We report here a case of a 73-year-old male patient with a progressive, low-grade febrile confusional syndrome 20 days after receiving the first dose of BNT162b2 vaccine against SARS-CoV-2. Evidence indicative of the underlying condition by an extensive panel of imaging (brain magnetic resonance imaging, computed tomography and digital subtraction angiography), laboratory (complete blood count, biochemistry, coagulation, tests for autoimmune or infectious disorders, tumor markers, hormonal levels, cerebrospinal fluid analysis) and electrodiagnostic tests were scarce, and mainly non-specific. Sequential neuroimaging revealed the appearance of extensive T2 lesions (signs of gliosis) along with multiple hemorrhagic lesions at various cortical sites. The patient was treated with corticosteroids, discontinued due to severe adverse effects, and subsequently with sessions of plasmapheresis and monthly intravenous administration of cyclophosphamide. Considering the rapid aggravation of the patient's neurological status, the MRI findings of cortical lesions and the lack of response to any treatment, a biopsy of a frontal lobe lesion was conducted, confirming the presence of confluent, inflammatory-edematous lesions with scattered areas of necrosis and hemorrhage, and ultimately areas of demyelination, thus confirming the diagnosis of AHLE. After more than 5 months of hospitalization the patient was transferred in a primary care facility and remained in a permanent vegetative state until his death, more than 2 years later.

## 1 Introduction

Acute hemorrhagic leukoencephalitis (AHLE), is a rare inflammatory disease, affecting mostly the cerebrum, and less commonly other parts of the central nervous system (CNS) [1]. It was first described by Weston Hurst in 1941 and is considered being a severe demyelinating disorder and a rare variant of acute disseminated encephalomyelitis [2]. The histopathologic profile of AHLE is mainly characterized by rapidly progressive and symmetrical, multifocal inflammatory lesions, associated with acute edematous necrosis and hemorrhage, especially at the perivascular areas of the CNS [3]. From an etiological/ pathophysiological point of view, the nature of AHLE is more obscure: an autoimmune process against components of the CNS, initiated due to cross reactivity to viral antigens, has been hypothesized, since many patients develop the condition soon after having recovered from viral infection or undergone vaccination [4].

The diagnosis of AHLE remains extremely challenging due to the rarity of the disease and the fact that its symptoms/ signs are indistinguishable from many other neurological disorders. AHLE usually affects male, adult individuals, and its clinical course usually involves rapid neurological decline, eventually leading to coma and death [5]. The prognosis of AHLE is poor and mortality was reported to be as high as 70 % in the past. Nowadays, intensive immunosuppression [6,7] protocols are being followed, which has improved the therapeutic outcome (with up to 25 % of the cases achieving a partial recovery), but disease morbidity still approaches 50 % [8].

**Fig. 1:**
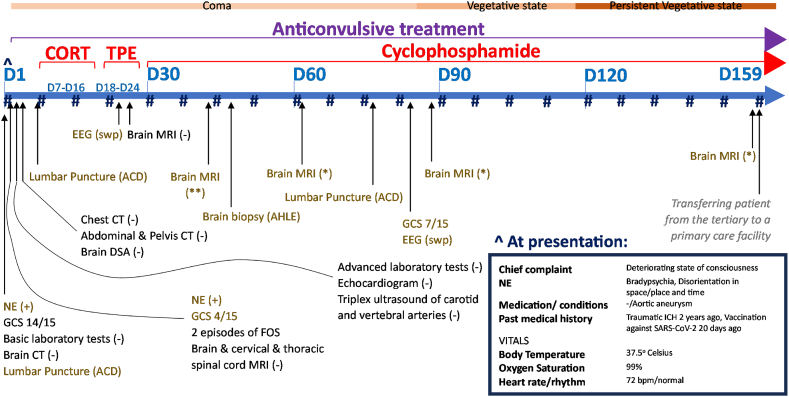
Overview of the course of the case from admission to the tertiary healthcare facility until being transferred to a primary healthcare facility. The patient remained hospitalized for 159 days (D1 to D159). During D2 he fell into a coma, only to pass into a vegetative state about 3 months later (D83), a state that holds until today. Along the timeline presented, the reader can see the different diagnostic procedures and therapeutic interventions applied, as well as other medically important pieces of information. Since D2 the patient was administered intravenous (iv) anticonvulsive treatment. For 9 days (between D7-D16) the patient was receiving iv synthetic glucocorticoids (CORT). Within a period of 1 week (D18-D24) the patient underwent 4 session of therapeutic plasma exchange (TPE). Since D30, the patient started with iv infusions of cyclophosphamide, once every month. #: SARS-CoV-2 detection via real time polymerase chain reaction from nasopharyngeal swabs (on a weekly basis, all result came out negative), (−): findings within the physiological spectrum, (+) abnormal findings, (**): hemorrhagic lesions, leptomeningeal enhancement & prominent sites of gliosis, (*): hemorrhagic lesions, gradual attenuation of the leukoencephalopathy, ACD: albuminocytologic dissociation, AHLE: acute hemorrhagic leucoencephalitis (diagnosis confirmed), bpm: beats per minute, CT: computed tomography, DSA: digital subtraction angiography, EEG: electroencephalography, FOS: focal onset seizures, GCS: Glasgow coma scale, ICH: intracerebral hemorrhage, MRI: magnetic resonance imaging, NE: neurological examination, swp: slow wave predominance.

We present this clinical case report (Fig. 1), aiming at creating awareness on AHLE, the challenges surrounding its differential diagnosis and therapeutic strategies, as well as debating on the potential relationship between AHLE and the vaccination against SARS-CoV-2.

## 2 Case presentation

A 73-year-old Caucasian male was brought to our emergency department by his family (wife and son) complaining about low-grade fever and a fluctuating, but gradually deteriorating state of consciousness which attracted the attention of his relatives over the last week. The patient was not receiving any kind of medication. The patient was diagnosed with an aneurysm of the abdominal aorta (around 4.5 cm in diameter) a few years ago. The patient had a medical history of a traumatic intracerebral hemorrhage of the right parieto-occipital lobe (two years before), which the relatives of the patient were not able to provide further details on. He was also vaccinated against SARS-CoV-2 (1st dose of BNT162b2) approximately twenty days prior symptom onset. Finally, according to his wife, the patient was not living with or in the proximity of (any kind of) animals. No family history of any chronic conditions was reported.

Initial clinical examination revealed a Glasgow coma scale (GCS) score of 14/15 (Eye opening response/e:4, Best verbal response/v:4, Best motor response/m:6) (patient was confused and disoriented but able to cooperate with the examiner and follow simple commands) with no signs of meningism or skin rush and no evident focal neurological signs (neither long tract signs nor signs of cranial nerve involvement). Tendon reflexes appeared normal as well. Routine blood tests, including complete blood count, coagulation, biochemistry, vitamin B12 and folic acid, hormone levels, vitamin levels, inflammatory markers and basic immunological tests, showed values within normal range. Brain computed tomography (CT) revealed no substantial findings aside a hypo-dense, right, parieto-occipital lesion (old hemorrhage, known from the patient's medical history) and chronic microvascular leukoencephalopathy. The patient also provided brain magnetic resonance (MR) images that were captured at an outpatient basis two days before his admission, which also only highlighted the area of the old intracerebral hemorrhage.

The lack of any laboratory and imaging findings, the non-specific, declining level of consciousness of the patient, in conjunction with the lack of signs of any recent focal neurological lesion, prompted the investigation of a large number of potential causes as part of the differential diagnostic process, including (para)neoplastic, inflammatory/ demyelinating, infectious, metabolic, toxic, and neurodegenerative diseases. Specifically, autoimmune encephalitis, infectious (viral/bacterial) encephalitis, autoimmune vasculitis, Creutzfeldt-Jakob disease, multiple sclerosis, and Devic's disease (Neuromyelitis Optica), John Cunningham virus encephalopathy, Bickerstaff encephalitis, acute disseminated encephalomyelitis, paraneoplastic syndromes, and neurovascular disorders. [Some of those disorders were introduced into the differential diagnosis list a few days/weeks later, after the sequential neuroimaging investigation, see below.]

During the first 24 hours of hospitalization, the clinical status of the patient deteriorated to a GCS score of 4/15 (e:1, m:1, v:2), left hemiparesis, choreoathetosis, gaze deviation to the right, and 2 episodes of focal onset seizures involving the left upper limb. Anticonvulsive treatment was initiated with intravenous (iv) valproate sodium that was later changed, due to lack of efficacy, to double anticonvulsive therapy with iv levetiracetam 1000 mg twice daily and lacosamide 100 mg (and later on 150 mg) twice daily, resulting in full control of the seizures. Blood samples were sent for laboratory testing against infectious agents, autoantibodies and tumor markers without eliciting any useful findings. Initial lumbar puncture revealed an albuminocytologic dissociation (normal white blood cell count but elevated protein levels), which was reconfirmed in a second lumbar puncture a week later, and a third one 2.5 months later (Table 1). Other tests of the cerebrospinal fluid (CSF) for oligoclonal bands, detection of anti-myelin oligodendrocyte glycoprotein (with cell-based assay methods) and other autoantibodies, markers of Creutzfeldt Jacob disease and polymerase chain reaction-based testing against an extensive panel of viruses (including John Cunningham virus), as well as tropheryma whipplei, were negative. Furthermore, extensive blood screening, including NMO, GQ1b and paraneoplastic antibodies did not elicit any notable findings, except for a mild elevation of titin antibodies, usually related to myasthenia gravis [10], thus not compatible with the patient's neurological profile.

**Table 1:** Findings from basic cerebrospinal fluid cell count and biochemical analysis. Normal values are for white blood cells 0–5 cells/mm^3^, for glucose 60–70 % of blood glucose, for protein 15–50 mg/dL and for lactate dehydrogenase (LDH) < 45 IU/L.

	DAY 1	DAY 7	DAY 76
**White Blood Cells**	4 mm^−3^	0 mm^−3^	1 mm^−3^
**[Glucose]**	75 mg/dL	110 mg/dL	63 mg/dL
**[Protein]**	233 mg/dL	227 mg/dL	294 mg/dL
**[LDH]**	21 IU/L	40 IU/L	59 IU/L

**Fig. 2:**
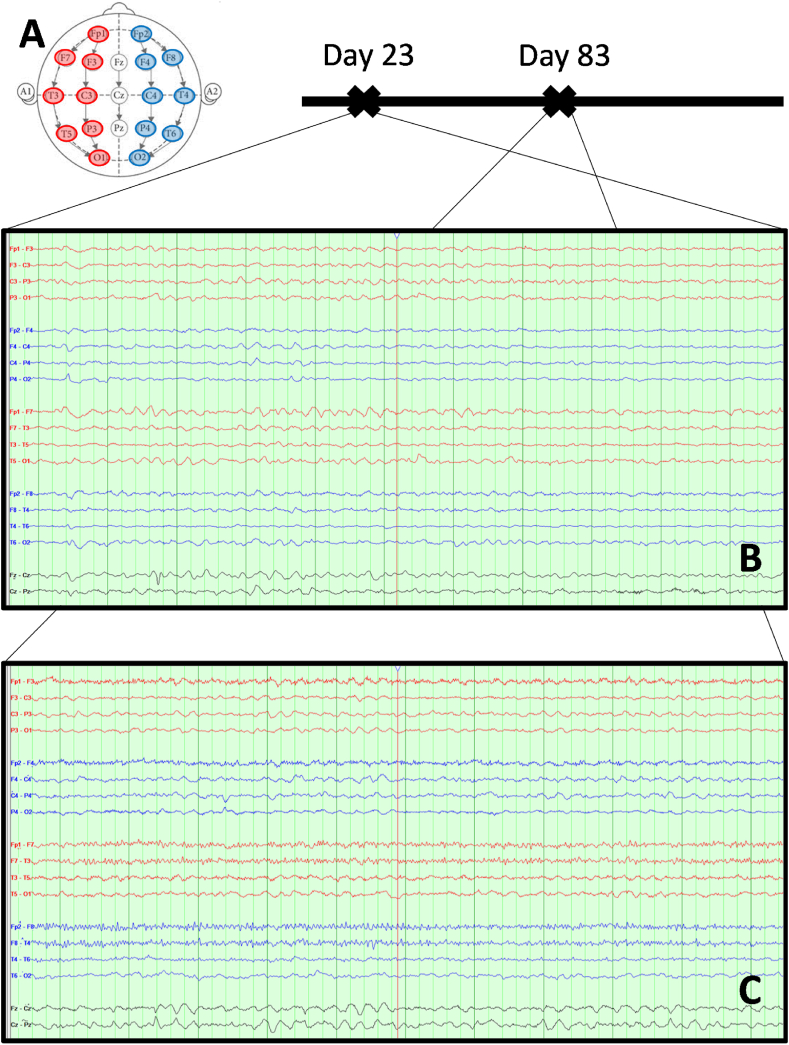
**Electroencephalogram (EEG) of the patient at two different timepoints.** (A) The International 10–20 system has been followed apply the location of scalp electrodes in the context of the EEG exam. (B) EEG at day 23 post-hospitalization. Νο epileptiform activity was observed. Note the generalized predominance of low frequency and low amplitude (delta and theta) waves, which correlate with reduced neural activity. (C) EEG at day 23 post-hospitalization. Improved neural activity, mainly in the frontal regions.

**Fig. 3:**
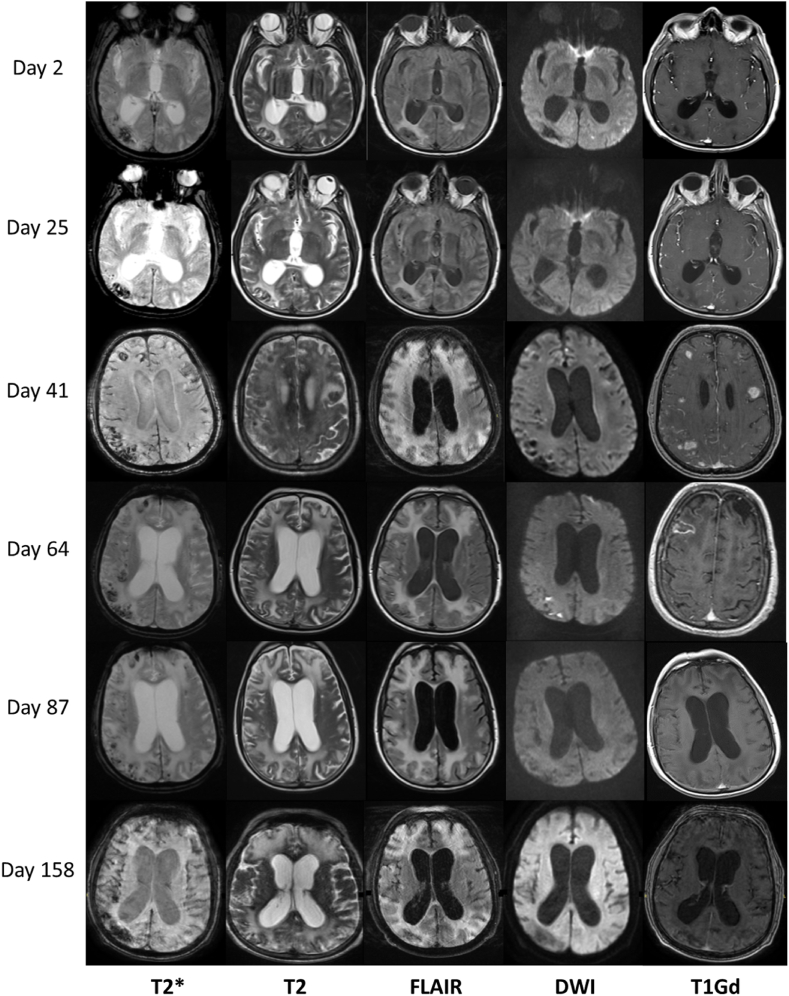
Longitudinal brain imaging findings at certain days post-hospitalization. Left column (T2* sequences): hypointense signal, indicative of hemorrhagic lesions, observed in the right temporooccipital and parietooccipital lobe, as well as left frontal lobe. Second and third columns (T2 and FLAIR sequences respectively): sites of gliosis (hyperintense signal) initially observed in the right parietooccipital lobe, gradually increasing in size and spreading to both cerebral hemispheres (indicative of an leukoencephalopathy), at the level of corona radiata, reaching the maximum exacerbation on the 41st day. Gradual attenuation of the leukoencephalopathy in the subsequent images, although diffuse sites of gliosis remain in the frontal and parietal lobes. There is a gradual increase in the volume of the ventricular system, in the context of brain atrophy. Fourth column (DWI sequences): no restriction in water diffusion is observed but two small focal sites in the right parietal lobe on the 64th day most likely of hemorrhagic origin. Last column (gadolinium enhanced T1 sequences): subacute, focal hemorrhagic sites, giving an increased signal intensity in the frontal and parietal lobes bilaterally, and in the right parietooccipital lobe on the 41st day, combined with right-sided leptomeningeal enhancement. Pathologically increased signal intensity in an area of the right frontal lobe on the 64th day (most likely due to brain biopsy). FLAIR: fluid-attenuated inverse recovery, MRI: magnetic resonance imaging, DWI: diffusion-weighted imaging.

Electroencephalography (Fig. 2A) was also performed and was indicative of encephalopathy, with absence of alpha rhythm, and widespread theta and delta activity, with no epileptiform discharges (Fig. 2B and C). A brain digital subtraction angiography (DSA) was negative for primary CNS vasculitis. Repeated brain MRI scans, over a period of 3 months (Fig. 3), progressively showed cortical hypointense T2*-weighted signal, indicative of hemorrhagic lesions, subcortical hyperintense T2-weighted signal, indicative of gliosis and focal hemorrhagic sites of increased gadolinium-enhanced T1-weighted signal.

After the negative laboratory tests, which were unsupportive of any possible infectious, metabolic, or neoplastic causes, intravenous corticosteroids at high doses (iv methylprednisolone 1000 mg/day for 7 days followed by prednisolone 25 mg twice daily) were initiated, and patient showed a minimal improvement of his GCS score (spontaneous opening of eyes). Nevertheless, at day 9 of glucocorticoid administration, treatment had to be stopped due to severe gastrointestinal bleeding, which was attributed to adverse effects of high doses of corticosteroids. Subsequently, the patient underwent 4 sessions of therapeutic plasma exchange, followed by monthly iv infusions of cyclophosphamide 1000 mg with only minimal improvement of his neurological status (GCS 7, with e:4, v:2, m:1).

**Fig. 4:**
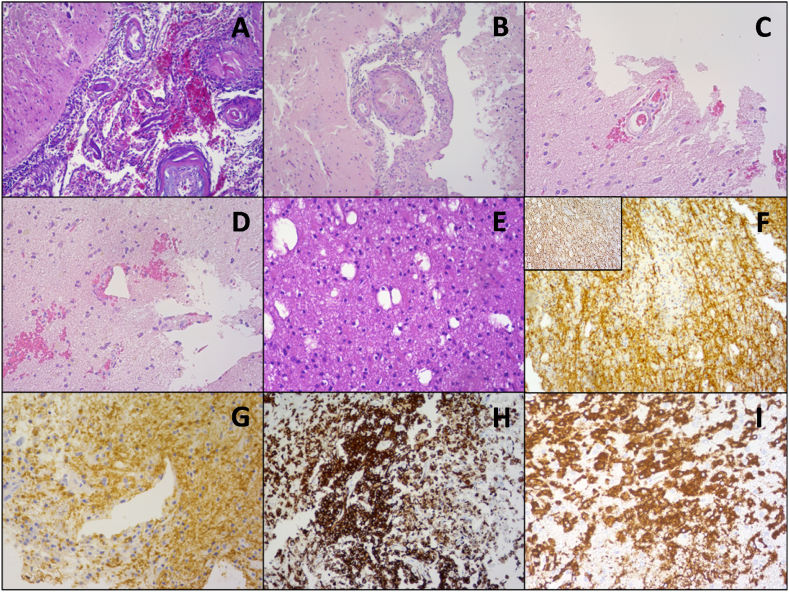
**Immunohistochemical analysis of a brain biopsy sample.** [A] Hematoxylin-Eosin (H&E) stain, ×100: perivascular hemorrhages and mild inflammatory infiltrates in the leptomeninges. [B] H&E stain, ×100: perivascular inflammatory infiltrates consisting of macrophages and lymphocytes. [C] and [D] H&E stain, ×200: perivascular hemorrhages. [E] H&E stain, ×200: glial tissue with cellular edema. [F] Myelin-Basic Protein (MBP) stain (brown), ×100: there are multiple small-sized areas of demyelination (inset: neurofilament stain with focal loss of axons). [G] MBP stain (brown), ×200: areas of perivascular demyelination. [H] CD163 stain (brown), ×100: perivascular monocytic focus. [I] CD163 stain (brown), ×200: parenchymal monocytic nodules. (For interpretation of the references to colour in this figure legend, the reader is referred to the Web version of this article.)

Considering the non-specific results of the paraclinical evaluation, the rapid deterioration of the patient's neurological status and the proximity of the brain lesions to the cerebral cortex, a biopsy was conducted on a right frontal lobe lesion, in collaboration with the neurosurgical unit of the hospital, to confirm the diagnosis. The histopathological evaluation of the samples confirmed the presence of perivascular hemorrhages and mild inflammatory infiltrates in the leptomeninges and the parenchyma (Fig. 4A–D), glial tissue with cellular edema (Fig. 4E), multiple small-sized areas of inflammatory demyelination, both parenchymal (Fig. 4F) and perivascular (Fig. 4G), as well as perivascular microglial foci and parenchymal microglial nodules (Fig. 4H and I), leading to the diagnosis of acute hemorrhagic leukoencephalitis [9]. After more than 5 months of hospitalization, the patient was transferred in a primary care facility, and he remained in a permanent vegetative state for over 2 years before passing away, without volitional control of movement, but with spontaneous opening of his eyes and a circadian alternation between sleep and awake states.

## 3 Discussion

We describe a rare case of AHLE, temporally associated with vaccination against SARS-CoV-2, involving a 73-year-old male patient with no prior medical history of chronic neurological disease, who sub-acutely exhibited a deteriorating level of consciousness, eventually leading to coma. After extensive screening for a variety of neurological disorders, the possibility of AHLE was suspected due to the evolution and extent of T2-weighted lesions and occurrence of multiple focal hemorrhagic sites (contrast-enhanced T1-weighted signal) in the brain MRI over a period of weeks, followed by a brain biopsy, which was decisive for the final diagnosis. Treatment included the iv administration of corticosteroids, sessions of plasmapheresis and finally sessions of iv administration of cyclophosphamide. Unfortunately, the clinical status of the patient did not improve notably, and the patient remained in a permanent vegetative state over 2 years after the diagnosis was confirmed, until he passed away. The non-specific neurological symptoms of the condition, the scarcity of laboratory findings, the asynchronous appearance of inflammatory/ demyelinating and hemorrhagic lesions in the brain MRI, as well as the poor response of the patient to different treatment protocols made the management of AHLE a real challenge.

Our patient presented many of the typical clinical/ laboratory/ radiological and histopathological features of AHEM [11,12] such as a rapidly progressive encephalopathy, focal neurological deficits, elevated protein in CSF, bilateral, confluent, inflammatory-edematous lesions of the cerebrum with scattered areas of necrosis and hemorrhage, and ultimately areas of demyelination in biopsy speciments. Finally, the patient was recently vaccinated against SARS-CoV-2 with the mRNA vaccine BNT162b2. A very limited number of AHEM cases have been reported following vaccination against SARS-CoV-2, four with the ChAdOx1 vaccine [13,14], and only one with BNT162b2 vaccine [15] (Table 2). Contrary to that latter case, involving the development of AHLE 2 days after receiving the 2nd dose of the vaccine, our patient developed AHLE 20 days after receiving the 1st dose of the vaccine.

**Table 2:** Synopsis of the previously reported cases of AHLE post-vaccination against SARS-CoV-2 in the literature.

Type of vaccine (days before symptom onset)	Sociodemographics & medical history	Diagnostic tests indicating pathology	Brain biopsy?	Therapeutic approach	Outcome after admission
1st dose ChAdOx1 (2) [13]	61-year-old male, hypothyroidism and polymyalgia rheumatica	Brain CTA, Brain MRI	–	High dose iv glucocorticoids, plasma exchange	Persistent vegetative state after 98 days
1st dose ChAdOx1 (9) [13]	25-year-old female	Brain MRI, CSF analysis	–	High dose iv glucocorticoids, plasma exchange	Persistent paraplegia after 42 days
1st dose ChAdOx1 (9) [13]	55-year-old female	Brain MRI, CSF analysis	+ (a)	High dose iv glucocorticoids	Coma and death after 39 days
1st dose ChAdOx1 (14) [14]	28-year-old female	Brain MRI, CSF analysis	–	High dose iv glucocorticoids	Significant improvement following 5-day treatment and full recovery at 84 days
2nd dose BNT162b2 (2) [15]	53-year-old male, rheumatoid arthritis (under treatment with methotrexate & etanercept)	Brain MRI, CSF analysis	+ (b)	High dose iv glucocorticoids, dialysis	Persistent vegetative state and death after 120 days
1st dose of BNT162b2 (This case)	73-year-old male, history of traumatic intracerebral hemorrhage and aneurysm of the abdominal aorta	Brain MRI, EEG, CSF analysis	+	High dose iv glucocorticoids, plasma exchange, cyclophosphamide	Permanent vegetative state (the patient passed away more than 2 years later).

CSF: cerebrospinal fluid, CTA: computed angiography, EEG: electroencephalography, iv: intravenous, MRI: magnetic resonance imaging.

a Brain biopsy revealed perivascular immune cell infiltrates, including neutrophilic granulocytes, tissue edema, and perivascular hemorrhage.

b Brain biopsy revealed perivascular infiltrates of macrophages and small CD3^+^ lymphocytes, signs of former hemorrhage and perivascular demyelination.

Although many reports support the hypothesis of an autoimmune process, following COVID-19 [16,17], other infections [18] and vaccination [19], as a trigger factor of the disorder, we cannot provide convincing evidence linking the recent vaccination of the patient with the development of the condition. The association between the vaccination and the condition arose as a consequence of the temporal proximity of the two events, which made an impression to the relatives of the patient, and consequently decided to stress that out when recording the medical history of the patient. Given the vast numbers of vaccinated individuals worldwide, the occurrence of AHLE in the post-vaccination window may be purely coincidental, and actually the most probable scenario. Nevertheless, one should consider the responsibility of the biomedical community to provide reports, like this one, when a novel vaccine or therapeutic agent is granted permission for wider distribution, and indeed similar reports have been published in 2021 and 2022. In this context, it is finally worth noting that in 3 out of 5 previously reported AHLE cases in temporal correlation with the vaccination against SARS-CoV-2 (Table 2), no biopsy has been received, and thus the diagnosis of AHLE has not been confirmed.

## 4 Conclusion

AHLE is a devastating, sub-acutely evolving neurological condition, which frequently leaves patients into a comatose state (if not fatal), showing limited and non-specific laboratory markers of disease, mainly the progressive appearance and wide subcortical expansion of T2 lesions. This usually creates the necessity for taking a brain biopsy to confirm the diagnosis. Its inflammatory nature prompts clinicians to use aggressive anti-inflammatory treatment strategies, although the prognosis of the condition remains poor. In this case, a 73-year-old Caucasian male developed AHLE in temporal association with his first dose of BNT162b2 vaccination against SARS-CoV-2. So far, a very limited number of AHLE cases have been reported in the literature to be temporally associated with vaccination against SARS-CoV-2, and this is the first case involving the first dose of BNT162b2. Despite the fact that many reports support the hypothesis of an autoimmune process causing AHLE, in response to triggering events, including vaccination, there are yet no convincing evidence linking the disease with vaccines against SARS-CoV-2.

## Ethics declarations

All procedures performed were under the institutional and national research committee's ethical standards and the 1964 Helsinki declaration, later amendments and comparable ethical standards.

Informed consent was obtained from the close relatives of the participant included in the study.

## Funding

No funding supported this work.

## Data availability statement

Data and original images in the current study are available from the corresponding author on reasonable request. The data associated with this report have not been deposited into any publicly available repository, because the authors can confirm that all relevant data are included in the article and/or its supplementary information files.

## CRediT authorship contribution statement

**Konstantinos Kalafatakis:** Writing – review & editing, Writing – original draft, Supervision, Investigation, Conceptualization. **Anna Margoni:** Writing – original draft, Investigation, Data curation. **Maria-Eleni Liakou:** Writing – original draft, Investigation, Data curation. **Christos Stenos:** Writing – original draft, Investigation, Conceptualization. **Panagiotis Toulas:** Writing – review & editing, Visualization, Software, Methodology, Formal analysis, Data curation. **Penelope Korkolopoulou:** Writing – review & editing, Visualization, Resources, Methodology, Formal analysis. **Eleftheria Lakiotaki:** Writing – review & editing, Visualization, Resources, Methodology, Formal analysis. **Spiridon A. Lafazanos:** Validation, Resources, Investigation. **Katerina Zekiou:** Validation, Investigation. **Panagiota Kardara:** Validation, Supervision, Investigation. **Aspasia Terentiou:** Validation, Supervision, Investigation. **Georgios Nikolaou:** Supervision, Project administration, Investigation, Conceptualization. **Georgios Stouraitis:** Supervision, Project administration, Investigation, Conceptualization.

## Declaration of competing interest

The authors declare that they have no known competing financial interests or personal relationships that could have appeared to influence the work reported in this paper.
